# Effects of acute aerobic exercise on arterial stiffness in transgender men

**DOI:** 10.3389/fphys.2023.1294284

**Published:** 2023-10-31

**Authors:** Mizuki Yamada, Hyunjun Gam, Nodoka Ikegami, Yuriko Nishikawa, Akira Ishikawa, Akiko Funaki, Tomoka Matsuda, Kayoko Kamemoto, Yuto Hashimoto, Takanobu Okamoto, Hiroki Yamazaki, Hirotoshi Tanaka, Mikako Sakamaki-Sunaga

**Affiliations:** ^1^ Graduate School of Health and Sport Science, Nippon Sport Science University, Tokyo, Japan; ^2^ Research Fellow, Japan Society for the Promotion of Science (JSPS), Tokyo, Japan; ^3^ Department of Physical Education, Yongin University, Gyeonggi, Repulic of Korea; ^4^ Graduate School of Sport Sciences, Waseda University, Saitama, Japan; ^5^ Department of Judo Therapy, Teikyo University of Science, Yamanashi, Japan; ^6^ Department of Sport Sciences and Research, Japan Institute of Sports Sciences (JISS), Tokyo, Japan; ^7^ Department of Exercise Physiology, Nippon Sport Science University, Tokyo, Japan; ^8^ Research Institute for Sports Science, Nippon Sport Science University, Tokyo, Japan; ^9^ Department of Internal Medicine, Teikyo University School of Medicine, Tokyo, Japan; ^10^ Department of Rheumatology, Kitasato University Kitasato Institute Hospital, Tokyo, Japan

**Keywords:** female-to-male (FtM), sex hormone replacement therapy (HRT), low-density lipoprotein cholesterol (LDL-C), pulse wave velocity (PWV), treadmill exercise, transgender health

## Abstract

Testosterone replacement therapy (TRT) in transgender men (TM) results in side effects such as elevated triglycerides and increased arterial stiffness. Exercise may be useful to ameliorate such effects, but no studies have examined the effects of acute aerobic exercise in TM. This study aimed to investigate the effects of acute aerobic exercise on arterial stiffness in TM. Thirty-six participants were included, comprising 12 TM (duration of TRT: 57.4 ± 30.3 months), 12 males and 12 females. All participants performed acute aerobic exercise on a treadmill at 50% heart rate reserve for 30 min. Arterial stiffness as measured by brachial-ankle pulse wave velocity (baPWV) was measured before exercise (Pre), 30 min after exercise (Post30), and 60 min after exercise (Post60). Serum sex hormone levels, and serum lipid profile were determined only before exercise. Serum low-density lipoprotein cholesterol (LDL-C) levels before exercise were significantly higher in TM than in males or females (males: *p* < 0.01; females: *p* < 0.05). At all points, baPWV in TM was significantly higher than in females (*p* < 0.05) and significantly lower than in males (*p* < 0.05). However, when comparing changes in baPWV over time in each group, significant decreases in Post30 and Post60 were seen in males compared to Pre (both *p* < 0.05), but no significant change after aerobic exercise was seen in TM or females. These results suggest that acute aerobic exercise yield different effects in TM than in males, but is unlikely to reduce arterial stiffness in TM receiving TRT.

## 1 Introduction

Transgender men (TM) are defined as individuals who are assigned female gender at birth and who later wish to change their sex to male (American Psychiatric Association, 2013). Such individuals may choose to undergo testosterone replacement therapy (TRT) if they wish to change their sex. However, TRT leads to various side effects resulting from a decrease in the female hormone estradiol (E_2_) and an increase in the male hormone testosterone (Irwig, 2017). TM who undergo TRT reportedly show lower E_2_ and higher testosterone levels than females (Van Caenegem et al., 2012). TM also initiates TRT, which results in E_2_ and testosterone levels similar to those in males. These changes in sex hormone levels worsen blood lipid profiles by decreasing high-density lipoprotein cholesterol (HDL-C) and increasing low-density lipoprotein cholesterol (LDL-C) and triglyceride (TG) levels. Such changes are associated with the development of atherosclerosis (Streed et al., 2017; Velho et al., 2017), which increases the risk of cardiovascular diseases.

In both sexes, estradiol helps improve endothelial function by relaxing vascular smooth muscles and increasing nitric oxide (NO) production (Stanhewicz et al., 2018). In females, arterial stiffness decreases due to increased E_2_ levels after adolescence (DuPont et al., 2019). Additionally, arterial stiffness was lower in females than in males, which suggests that E_2_ may significantly influence sex-specific differences in arterial stiffness. In contrast, testosterone reportedly increases arterial stiffness in females by inhibiting NO production from vascular endothelial cells and decreases arterial stiffness in males by protecting against the onset of atherosclerosis (Stanhewicz et al., 2018). Together, these observations suggest that increased arterial stiffness is a direct effect of changes in sex hormone levels. The effects of sex hormones on arterial stiffness also differ between male and female patients. It has been found that TM undergoing TRT shows higher arterial stiffness compared to untreated TM, and transgender women treated with estrogen have lower arterial stiffness (Emi et al., 2008; Sharula et al., 2012). This finding further suggests that sex hormone changes during TRT not only worsen blood lipid profiles but also increase arterial stiffness.

The World Health Organization guidelines for cardiovascular disease prevention indicate that exercise improves blood pressure (BP) and lipid profiles (WHO, 2007). Acute aerobic exercise (AE) also reportedly reduces post-exercise arterial stiffness (Saz-Lara et al., 2021). Among young males and females, arterial stiffness of the lower extremities was reduced in males 60 min after 45 min of treadmill exercise at 70% heart rate reserve (Sun et al., 2020). Baseline arterial stiffness has also been suggested to be associated with sex-specific in the effects of acute exercise (DuPont et al., 2019). Lower E2 and higher testosterone levels in the TM may increase arterial stiffness, resulting in a higher baseline arterial stiffness. Thus, in turn, could potentially influence the effects of acute AE, although the exact relationship in this context remains unclear.

The present study examined the effects of acute AE on arterial stiffness in TM, comparing the findings with young males and females. We hypothesized that TM individuals undergoing TRT have lower E_2_ levels and higher testosterone levels than females or males, resulting in higher arterial stiffness. Furthermore, arterial stiffness in the TM, unlike in females, declined after acute AE, as seen in males. The results of this study contribute to understanding the exercise requirements for chronic AE training.

## 2 Materials and methods

### 2.1 Participants

This study recruited 12 participants each from TM (26.9 ± 4.2 years), male (24.7 ± 2.4 years), and female (24.9 ± 2.8 years) categories. None of the participants habitually exercised >2 days/week, had any history of obesity (BMI >30 kg/m^2^), hypertension (BP > 140/90 mmHg), or were smokers. In accordance with the Declaration of Helsinki, all participants were provided with verbal and written explanations of the ethical considerations of the study and provided written informed consent prior to enrolment (Harriss et al., 2022). This study was approved by the Institutional Ethics Committee for Human Experiments (approval no. 021-H158).

All TM were individuals who had been on TRT for at least 12 months (Berra et al., 2006; Wierckx et al., 2014; Quirós et al., 2015), taking into account changes in blood lipid profile due to different durations of treatment (57.4 ± 30.3 months [min–max: 18–99 months]). Testosterone was administered at 125 or 250 mg once every 2–3 weeks or 1,000 mg once every 4–5 months. Furthermore, measurements were taken 13–15 days after the most recent testosterone dose to account for sex hormone fluctuations during the 2–3 weeks of TRT (Nakamura et al., 2013). Six TM participants who underwent TRT underwent sex reassignment surgery. Blood profiles of female participants were assessed during the luteal phase (23.4 ± 3.1 days of the menstrual cycle) when female hormones (E_2_ and progesterone [P_4_]) were high. We questioned female participants about their menstrual cycles in the last 6 months to verify the presence of a normal menstrual cycle (25–38 days). We also confirmed the menstrual cycle phase using an ovulation test (DO-TEST; ROHTO Pharmaceutical Co., Osaka, Japan). Testing was performed continuously for seven consecutive days until a positive test was confirmed based on the urinary levels of luteinizing hormone (>30 mIU/mL). Moreover, blood P_4_ levels (>5.0 ng/mL) were analyzed to determine the luteal phase (Janse DE Jonge et al., 2019).

### 2.2 Experimental protocol

After visiting the laboratory, the height was measured using a stadiometer (YG-200; YAGAMI Inc., Aichi, Japan). Body composition was measured by a bioelectrical impedance analyzer (InBody 770; InBody Japan Inc., Tokyo, Japan). All participants were instructed to refrain from exercising and to avoid alcohol and caffeine for 24 h prior to starting the experiment. They were also instructed to maintain a normal diet and avoid excessive intake of fluids or high-fat meals (Wagganer et al., 2015). Blood samples were collected following a 12-h fasting period. Upon their visit to the laboratory between 08:30–09:30, body composition was measured using bioelectrical impedance analysis. All measurements were performed in a quiet, temperature-controlled room maintained at 23°C–25°C. The participants rested supine in the room for 30 min, after which the brachial-ankle pulse wave velocity (baPWV), brachial BP and heart rate (HR) were measured (PWV/ABI form: Omron Colin Co., Kyoto, Japan). Using the baPWV method, BP measurement cuffs wrapped around both the upper arms and ankles were inflated at low pressure (50 mmHg), and measurements were made using a volume pulse wave (Okamoto et al., 2019). Taken as the result for the right upper arm. Specifically, distance from the aortic valve opening to the upper arm (Lb) and the distance from the aortic valve opening to the ankle (La) were calculated using the following estimation formulae based on participants height:

$$\left(\text{Lb}\right)=0.2195\times \text{height }\left(\text{cm}\right)-2.0734$$

$$\left(\text{La}\right)=0.8129\times \text{height }\left(\text{cm}\right)+12.328$$

Finally, baPWV was calculated by dividing La-Lb by the time difference (ΔT) in the rise of the pulse wave between the upper arm and ankle. A single investigator performed all measurements, and the coefficient of variation (CV) for baPWV measurements was 2% ± 1%. Following baPWV measurements, blood was collected from the antecubital vein before exercise (Pre) using with a winged static-injection needle. Aerobic exercise was performed by fast walking or jogging for 30 min at moderate intensity on a treadmill (Biomill; S&ME, Tokyo, Japan). Each treadmill exercise session started with a 1 minute of warm-up. Subsequently, the treadmill speed was increased until the HR coincided with a 50% heart rate reserve (HRR) for 30 min (WHO, 2007; Garber et al., 2011; Zheng et al., 2015). The target HR was calculated using the Karvonen method ([HRmax (220–age)–HRrest] × %intensity) + HRrest) (Karvonen and Vuorimaa, 1988). During exercise, the HR was measured using an ECG monitor (BSM-3400; Nihon Kohden Co., Tokyo, Japan). The participants rested in the supine position for 60 min. The baPWV, BP, and HR were measured 30 min after exercise (Post30) and 60 min after exercise (Post60).

### 2.3 Blood analyses

Blood count data were analyzed for 11 females because hematological contractors were unavailable for the study. Venous blood samples were collected in vacuum tubes containing a serum separator. After coagulation at room temperature (20°C–22°C), serum was extracted by centrifugation at 3,000 rpm for 10 min at 4°C, stored separately at 4°C, and frozen at −20°C. Serum samples were analyzed for E_2_, P_4_, and testosterone (T) using a chemiluminescent immunoassay (E_2_: CV < 1.9%; P_4_: CV < 3.3%; T: CV < 2.8%). Free testosterone (FT) was measured using a radioimmunoassay, while TC, HDL-C, LDL-C, and TG levels were measured using by enzymatic methods (FT: CV < 9.0%, TC: CV < 0.7%, HDL-C: CV < 1.0%, LDL-C: CV < 1.4%, TG: CV < 1.3%). Plasma samples were collected in blood collection tubes containing EDTA-2K anticoagulant and refrigerated at 4°C. Red blood cells (RBC) and platelets were analyzed using electrical resistance detection, hemoglobin levels were determined through the sodium lauryl sulfate-hemoglobin method, and hematocrit was mesured using the RBC pulse wave height detection method (RBC: CV < 1.5%, platelets: CV < 4.0%, hemoglobin: CV < 1.0%, and hematocrit: CV < 1.5%). Whole blood was collected for glucose analysis and measured simultaneously with serum and plasma samples. Blood glucose levels were measured using a self-monitoring device (GLUCOCARD G Black; ARKRAY, Kyoto, Japan).

### 2.4 Statistical analysis

The sample size was determined using G*Power (version 3.1.9.7; Heinrich-Heine University, Düsseldorf, Germany). The primary endpoint under investigation was baPWV, and the potential variation in baPWV over time (Pre, Post30, Post60) among the three groups (TM, men, women) was calculated for statistical power using a two-way analysis of variance (ANOVA) with an estimated effect size of 0.25, α = 0.05, β = 0.8, consistent with previous studies (Okamoto et al., 2019). A minimum of 12 participants were included in the study in each group. All analyses were performed using the IBM SPSS Statistical for Windows, version 27.0 (IBM Corp., Armonk, N. Y., United States of America). Normality was assessed using the Shapiro–Wilk test, and homogeneity of variance was determined using the Levene’s test. E_2_, P_4_, FT, HDL-C, and TG levels were post-tested using the Kruskal–Wallis test to identify possible differences between the three groups. Post–hoc analysis with the Mann–Whitney U test and Bonferroni correction was performed to analyze the differences between groups. Changes in T, glucose, TC, LDL-C, glucose, RBC, hemoglobin, hematocrit, thrombocyte, and baPWV over time were obtained using one-way ANOVA, followed by Bonferroni post–hoc testing. A two-way ANOVA was conducted, considering both BP and baPWV of the group (TM group and male group, female group) × time (Pre and Post30, Post60). We also analyzed systolic blood pressure (SBP) as an additional covariate to eliminate its influence. The homogeneity of variance was assessed using Mauchly’s sphericity test, which confirmed the assumption of sphericity. Multiple comparisons were performed using the Dunn–Bonferroni post–hoc method. Effect sizes for the Mann–Whitney U test were calculated using z-values (*r* = z/√n), and one and two-way ANOVA were calculated using eta-squared (η^2^). Effect sizes were defined as small (*r* = 0.10, η^2^ = 0.01), medium (*r* = 0.30, η^2^ = 0.06), and large (*r* = 0.50, η^2^ = 0.14) (Cohen, 1998). The level of statistical significance was set at *p* < 0.05.

## 3 Results

### 3.1 Physical characteristics

The physical characteristics of the participants are summarized in Table 1. Height was significantly lower in the TM group and females than in males (*p* < 0.01). Body fat was significantly higher in the TM group and females than in males (*p* < 0.01). Furthermore, skeletal muscle mass was significantly lower in the TM group than in males (*p* < 0.01) and was higher in females (*p* < 0.01).

**TABLE 1:** Physical characteristics.

	TM (n=12)	Males (n=12)	Females (n=12)	*P*	η^2^	Post hoc comparisons
Height (cm)	161.3 ± 6.9	172.4 ± 6.8	157.4 ± 4.0	<0.01	0.55	Males > TM, Females**
Body weight (kg)	60.9 ± 6.7	65.4 ± 7.6	55.3 ± 6.2	<0.05	0.28	Males > Females**
BMI (kg/m^2^)	23.4 ± 2.1	22.0 ± 2.1	22.3 ± 2.2	0.25	0.81	-
Body fat (%)	23.7 ± 4.3	15.0 ± 4.3	27.3 ± 5.7	<0.01	0.56	Males < TM, Females**
Skeletal muscle mass (kg)	43.7 ± 4.4	52.4 ± 5.3	37.6 ± 2.5	<0.01	0.69	Males > TM, Females**
						TM > Females**

Values are expressed as mean ± standard deviation. TM: transgender men; BMI: body mass index.

**p<0.01.

### 3.2 Pre-exercise blood parameters

Pre-exercise blood analysis results are summarized in Table 2. E_2_ and P_4_ levels were significantly lower in TM patients than in females (E_2_: *p* < 0.01, *r* = 0.92; P_4_: *p* < 0.01, *r* = 1.04). Testosterone and FT levels were significantly higher in TM individuals than in females (T: *p* < 0.01; FT: *p* < 0.01, *r* = 0.81). However, sex hormone levels did not differ significantly between TM and males. Glucose levels were significantly lower in TM individuals than in males (*p* < 0.05). Total cholesterol was significantly higher in the TM group than in males (*p* < 0.01). Low-density lipoprotein cholesterol was significantly higher in TM than in males or females (males: *p* < 0.01, females: *p* < 0.05). RBC, hemoglobin, and hematocrit levels were significantly higher in TM individuals than in females (all *p* < 0.01). TG and thrombocyte counts showed no significantly differences between the three groups.

**TABLE 2:** Comparison of baseline blood collection results.

	TM (n=12)	Males (n=12)	Females (n=12)	*P*	η^2^	Post hoc comparisons
E_2_ (pg/mL)	12.0 [10.0–16.3]	14.5 [10.3–24.8]	160.5 [125.0–217.0]	<0.01^b^	-	TM, Males < Females**
P_4_ (ng/mL)	0.1 [0.1–0.1]	0.2 [0.1–0.2]	17.2 [12.2–19.2]	<0.01^b^	-	TM, Males < Females**
T (ng/mL)	5.9 [3.9–7.1]	6.0 [4.6–7.7]	0.3 [0.3–0.4]	<0.01^a^	0.75	TM, Males > Females**
FT (pg/mL)	13.1 [7.7–18.4]	15.1 [11.9–17.0]	1.0 [0.8–1.2]	<0.01^b^	-	TM, Males > Females**
TC (mg/dL)	197.5 [185.3–213.5]	162.0 [133.5–186.3]	183.0 [163.8–195.0]	<0.01^a^	0.28	TM > Males**
HDL-C (mg/dL)	56.5 [50.3–64.0]	50.5 [47.0–61.8]	66.0 [57.5–71.8]	<0.05^b^	-	-
LDL-C (mg/dL)	120.5 [105.3–141.0]	92.0 [67.3–115.5]	94.0 [87.8–108.8]	<0.01^a^	0.31	TM > Males, Females**^,^ *
TG (mg/dL)	75.5 [60.0–77.8]	71.5 [47.3–91.8]	58.5 [46.3–71.8]	0.28^b^	-	-
Glucose (mg/dL)	82.0 [79.5–87.5]	90.0 [85.0–95.0]	83.0 [80.0–86.5]	<0.01^a^	0.30	TM, Females < Males**^,^ *

	TM (n=12)	Males (n=12)	Females (n=11)	*P*	η^2^	Post hoc comparisons
RBC (×10^4^/μL)	513.0 [489.5–535.8]	498.5 [477.8–507.3]	434.0 [427.0–499.0]	<0.01^a^	0.32	TM, Males > Females**^,^ *
Hemoglobin (g/dL)	15.7 [14.6–16.1]	15.2 [14.6–15.6]	13.2 [12.6–14.4]	<0.01^a^	0.48	TM, Males > Females**
Hematocrit (%)	46.9 [46.0–48.2]	45.3 [44.1–46.7]	41.6 [39.7–43.9]	<0.01^a^	0.39	TM, Males > Females**
Thrombocytes (×10^4^/μL)	26.8 [21.1–30.6]	24.1 [21.0–25.8]	25.2 [20.6–26.8]	0.80^a^	0.01	-

Values are expressed as median [interquartile range]. TM: transgender men; E_2_: estradiol; P_4_: progesterone; T: testosterone; FT: free testosterone; TC: total cholesterol; HDL-C: high-density lipoprotein-cholesterol; LDL-C: low-density lipoprotein-cholesterol; TG: triglycerides, RBC: red blood cells.

^a^ Analysis of variance test.

^b^ Kruskal-Wallis test.

*p<0.05; **p<0.01.

### 3.3 Blood pressure and heart rate

Changes observed in BP and HR over time are summarized in Table 3. Systolic blood pressure (SBP), mean blood pressure (MBP) and pulse pressure (PP) at all time points were significantly lower in TM individuals than in males (SBP: *p* < 0.01; MBP: *p* < 0.05; PP: *p* < 0.01). Systolic blood pressure and PP were significantly lower at Post60 than at Pre- and Post30 in all groups (both *p* < 0.01). The MBP and HR were significantly higher at in Post30 than at Pre in all groups (MBP: *p* < 0.05; HR: *p* < 0.01).

**TABLE 3:** Changes in blood pressure and heart rate.

		Pre	Post30	Post60	Interactional		Group		Time	
					η^2^	*p*	η^2^	*p*	η^2^	*p*
SBP (mmHg)	TM	107.3 ± 5.4^**^	108.4 ± 4.5^**^	104.6 ± 6.0**^, ††, ‡‡^						
	Males	117.8 ± 11.8	117.1 ± 9.7	114.7 ± 11.5^††, ‡‡^	0.01	0.91	0.46	<0.01	0.19	<0.01
	Females	101.7 ± 5.3^**^	101.7 ± 5.8^**^	99.2 ± 6.3**^, ††, ‡‡^						
MBP (mmHg)	TM	78.1 ± 4.9^*^	80.4 ± 3.1*^, †^	77.3 ± 3.3*^, ‡‡^						
	Males	86.3 ± 9.6	87.9 ± 9.6^†^	84.2 ± 10.5^‡‡^	0.02	0.84	0.44	<0.01	0.20	<0.01
	Females	72.5 ± 5.0^**^	74.0 ± 6.4**^, †^	72.0 ± 4.8**^, ‡‡^						
DBP (mmHg)	TM	61.2 ± 5.4	62.1 ± 5.0	60.9 ± 5.7						
	Males	64.6 ± 8.1	65.0 ± 8.6	64.2 ± 8.2	0.03	0.77	0.19	<0.05	0.03	0.31
	Females	58.9 ± 4.3^*^	57.9 ± 5.3^*^	57.2 ± 3.7^*^						
PP (mmHg)	TM	46.1 ± 4.7^**^	46.4 ± 4.5^**^	43.7 ± 5.1**^, ††, ‡^						
	Males	53.3 ± 4.3	52.1 ± 2.8	50.5 ± 5.0^††, ‡^	0.04	0.54	0.55	<0.01	0.17	<0.01
	Females	42.7 ± 3.7^**^	43.7 ± 3.1^**^	42.0 ± 4.3**^, ††, ‡^						
HR (beats/min)	TM	56.7 ± 7.0	64.0 ± 7.7^††^	61.6 ± 6.4^††, ‡^						
	Males	60.8 ± 8.4	68.4 ± 9.8^††^	63.7 ± 10.4^††, ‡^	0.05	0.22	0.04	0.52	0.44	<0.01
	Females	60.3 ± 7.0	64.4 ± 7.5^††^	64.1 ± 7.7^††, ‡^						

Values are expressed as mean ± standard deviation. TM: transgender men; SBP: systolic blood pressure; MBP: mean blood pressure; DBP: diastolic blood pressure; PP: pulse pressure; HR: heart rate; Pre: before exercise; Post30: 30 min post-exercise; Post60: 60 min post-exercise.

*p<0.05; **p<0.01 vs. Males; ^†^ *p*<0.05; ^††^ *p*<0.01 vs. Pre; ^‡^ *p*<0.05; ^‡‡^ *p*<0.01 vs. Post30.

### 3.4 baPWV

**FIGURE 1:**
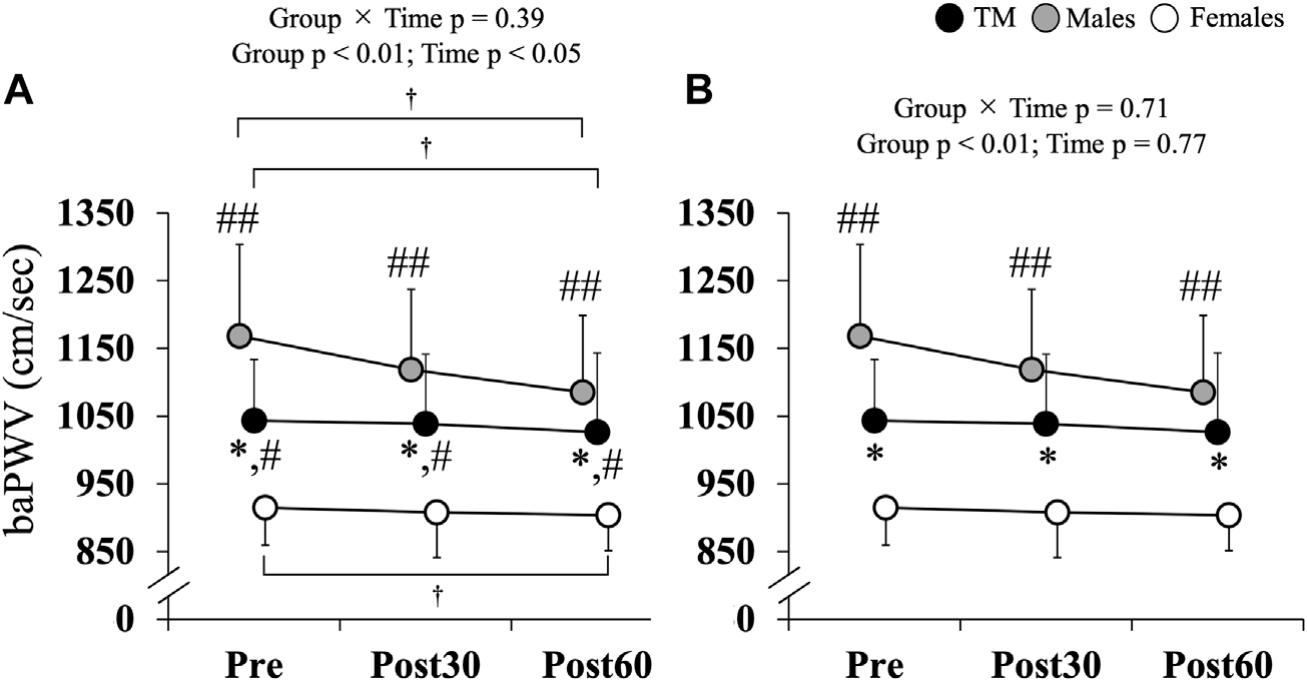
**(A)** Changes in brachial-ankle pulse wave velocity. **(B)** Changes in brachial-ankle pulse wave velocity. Data adjusted for SBP. Values are expressed as the mean ± standard deviation. baPWV: brachial-ankle pulse wave velocity; TM: transgender men; Pre: before exercise; Post30: 30 min after exercise; Post60: 60 min after exercise. *p < 0.05 vs. Males; #*p* < 0.05; ##*p* < 0.01 vs. Females; †*p* = 0.07 vs. Pre.

Changes in baPWV over time are shown in Figure 1, with baPWV significantly lower in TM (Pre, Post30 and Post60) than in males (Pre: TM 1,024.0 ± 107.4 cm/s vs. males 1,169.4 ± 127.4 cm/s; Post30: TM 1,018.7 ± 118.5 cm/s vs. males 1,126.6 ± 112.6 cm/s; Post60: TM 1,011.3 ± 122.4 cm/s vs. males 1,122.9 ± 103.7 cm/s; *p* < 0.05) and significantly higher than in females (Pre: 926.9 ± 54.5 cm/s; Post30: 919.3 ± 61.7 cm/s; Post60: 910.5 ± 73.2 cm/s; *p* < 0.05) at all time points (Group × Time, *p* = 0.34, η^2^ = 0.05; Group, *p* < 0.01, η^2^ = 0.49; Time, *p* < 0.05, η^2^ = 0.10). All groups showed lower values at Post60 than at Pre (*p* = 0.07). After correcting for SBP, the difference between the TM and females disappeared, and the values remained significantly lower in the TM than in males (*p* < 0.01). Furthermore, upon comparing the changes in baPWV over time in each group, a significant reduction was observed at Post30 and Post60 in males compared to Pre (both *p* < 0.05) (Table 4); however, no significant differences were observed in TM or females compared to pre-exercise levels.

**TABLE 4:** Changes in baPWV before and after exercise.

	Pre	Post30	Post60	*P*
TM	1024.0 ± 107.4	1018.7 ± 118.5	1011.3 ± 122.4	0.70
Males	1169.4 ± 127.4	1126.6 ± 112.6^†^	1122.9 ± 103.7^†^	<0.01
Females	926.9 ± 54.5	919.3 ± 61.7	910.5 ± 73.2	0.54

Values are expressed as mean ± standard deviation. TM: transgender men; Pre: before exercise; Post30: 30 min post-exercise; Post60: 60 min post-exercise.

^†^ *p*<0.05 vs. Pre.

## 4 Discussion

This study aimed to examine the effects of acute AE on arterial stiffness in TM individuals and compare these results with those of males and females. In this study, TM had lower E_2_ and higher T levels than non-TM individuals. To the best of our knowledge, these results represent the first evidence suggesting that pre-exercise serum LDL-C levels are significantly higher in TM than in males or females. Furthermore, baPWV was significantly lower in the TM group than in males and significantly higher in females at all time points. A significant reduction was observed in males at Post30 and Post60 compared to Pre; however, no significant changes were observed in the TM or females.

In the TM, sex hormone changes (such as decreased E_2_ and increased T) increase blood LDL-C levels. Hormonal responses to E_2_ and T in humans reportedly differ according to the presence of X and Y sex chromosomes (Arnold et al., 2017). In other words, the hormonal responses differed between males and females. The assumption underlying this study was that since TM did not have a Y chromosome, they would display different hormonal responses compared to males. Serum total and FT levels in postmenopausal females reportedly increase TC, LDL-C, and TG (Lambrinoudaki et al., 2006). In contrast, negative correlations of serum testosterone levels with TC and LDL-C levels have been reported in males (Simon et al., 1997). Thus, there are discernible sex-specific differences in the effect of testosterone on lipid profiles. A few months after initiating TRT, TM showed a decline in reproductive organ function, cessation of menstruation, and decreased serum E_2_ levels (Ahmad and Leinung, 2017). Reduced in serum E_2_ levels in TM have been shown to worsen blood lipid levels such as HDL-C and LDL-C (Streed et al., 2017; Velho et al., 2017). Serum LDL-C is higher in males than in females up to 40 years of age, but higher in females than in males after 50 years of age (Arai et al., 2005). Serum LDL-C level also reportedly increases in females who have undergone ovariectomy (Ikenoue et al., 1999). These differences in profile have been attributed to the increased serum LDL-C levels caused by decreased serum E_2_ levels due to menopause or ovariectomy. In females, serum E_2_ increases hepatic LDL receptor activity, which in turn increases LDL uptake from the blood into the liver, resulting in decreased serum LDL-C (Godsland, 2001). Lower E_2_ levels also decrease the number of LDL receptors in the liver, leading to decreased LDL uptake into the liver and increased production of lipoprotein lipase, resulting in the accumulation of LDL particles in the blood (Arca et al., 1994; Wakatsuki and Sagara, 1995). Based on these findings, TRT can be said to increase T in the TM, which in turn lowers E_2_ and increases serum LDL-C levels. Additionally, because the biological sex of the TM is female, the effects of sex hormones may manifest similarly to those in postmenopausal and ovariectomized females.

Age, blood lipid levels, and BP identified as factors that can increase arterial stiffness and contribute to atheroma progression, endothelial dysfunction, extracellular matrix accumulation, and plaque rupture (Kim and Kim, 2019). In this study, we investigated the impact of sex hormones that affect baPWV to determine the mechanisms that produce differences between TM in males and females. Males in this study demonstrated baPWV values similar to those in males in a previous study (Lu et al., 2023). In contrast, values in females were lower than those in the previous study, and values in the TM were similar to those in females in the previous study. In other words, the TM group in this study did not show a significant increase in arterial stiffness. The baPWV was significantly lower in the TM group than in males and significantly higher than in females at all time points. However, after correction for SBP, TM showed no significant difference when compared to females and significantly lower levels compared to males. In addition, to sex hormones, height has been cited as a factor contributing to sex differences in PWV, with shorter stature indicating a lower PWV (Duprez and Jacobs, 2012). Thus, females exhibit a lower PWV than males owing to their shorter stature. The TM in this study were shorter than the males. Furthermore, PWV increases in hypertensive cases due to decreased NO activity and endothelium-dependent vasodilatory responses resulting from the increased production of reactive oxygen species and other factors (Battelli et al., 2014; Gkaliagkousi et al., 2015). Thus, an elevated SBP was thus clearly associated with an increased PWV. In this study, height and SBP were lower in TM than in males, suggesting that the baPWV was significantly lower in TM than in males at all time points. In contrast, the baPWV was significantly higher in TM individuals than in female patients at all time points, primarily due to changes in sex hormones. Estrogen exerts vasodilatory effects in males and females (Stanhewicz et al., 2018). In contrast, testosterone inhibits NO production from vascular endothelial cells and causes impaired vasodilation in females but promotes vasodilation in males (Stanhewicz et al., 2018). In postmenopausal females, a high free androgen index (total testosterone/sex hormone-binding globulin) is associated with increased arterial stiffness (Lambrinoudaki et al., 2017). Arterial stiffness is reportedly higher in males with low serum testosterone levels (Vlachopoulos et al., 2014). This suggests that sex hormones directly alter arterial stiffness. Conversely, elevated LDL-C levels resulting from changes in sex hormone levels may also increase arterial stiffness. Elevated blood LDL-C levels cause LDL infiltration and stagnation under blood vessel. After macrophages take up oxidized LDL, cholesterol starts accumulating, resulting in arteriosclerosis (Hansson, 2005). However, given that the mean LDL-C level in the TM was 120 mg/dL, a focal atherosclerotic lesions was considered unlikely to have formed at this time. Therefore, baPWV in this study may have been higher in TM than in females because of TRT-induced changes in sex hormones. As mentioned earlier, SBP influences baPWV, and because no significant difference in SBP was evident between TM and females, there was likely no difference in baPWV when corrected for SBP. However, long-term elevated LDL-C levels in the TM or its further elevation can contribute to pathology. Further detailed studies on this topic are warranted. Atherosclerosis may occur even in young TM (20–30 years old); ,therefore, morphological assessment using ultrasonography and computed tomography is advisable.

In this study, baPWV decreased 60 min after acute AE in males but remained unchanged in TM and females. Aerobic exercise increases blood flow and shear stress by producing NO, which decreases arterial stiffness (Green et al., 2017). A previous study reported a decrease in arterial stiffness in males and no change in females at 60 min after treadmill exercise (Sun et al., 2020). This study found differences in lower extremity arterial stiffness after exercise due to sex-specific differences in the degree of vasodilation relative to the lower extremity muscles because of the greater lower extremity muscle mass in men (Sun et al., 2020). In this study, we found that TM had less skeletal muscle mass than males, which may explain why baPWV did not decrease after exercise. Furthermore, high blood levels of LDL-C in the TM may lead to increased atherosclerosis because of increased oxidized LDL (Hansson, 2005). High BP and high LDL-C levels attenuate the effects of AE (Zang et al., 2022). It is worth noting that the lack of an effect of exercise on arterial stiffness in young females is stated to be due to their lower baseline arterial stiffness compared to with young males, which limits the potential for arterial stiffness reduction through exercise (DuPont et al., 2019). To summarize, baPWV did not decrease after acute exercise in TM. This could be attributed to several factors, such as less muscle mass in the lower extremities in TM compared to males, which may not stimulate vasodilation after exercise, and counteraction of the effect of exercise by an increase in blood LDL-C. The baseline baPWV in the TM individuals was lower than that in males, suggesting a response similar to that in females, wherein exercise minimally suppressed the decline in arterial stiffness. This study examined the effects of acute exercise in males and females of the same age and under the same exercise conditions. The baPWV values in the TM differed from those in males and were similar to those in females. Previous studies have shown that the increase in acute exercise improves the effectiveness of exercise interventions (Okamoto et al., 2018). Therefore, interventional studies should be conducted in the future to examine these effects in depth.

Despite yielding novel and encouraging results, this study also had several limitations. Some TM in this study underwent sex reassignment surgery (uterine oophorectomy), while others did not. Sex reassignment surgery may affect blood lipid profiles and arterial stiffness in the TM because it lowers E_2_ (Pelusi et al., 2014; Defreyne et al., 2020). While further studies are needed, they were not considered problematic in this study because all TM participants had undergone TRT for at least 12 months, the number of days since the most recent testosterone administration was standardized, and sex hormone levels were taken into consideration. Additionally, the day before this experiment, the participants were instructed to consume their routine diet, as in a previous study; however, no dietary survey was performed. As dietary cholesterol intake is known to affect serum cholesterol levels (Clarke et al., 1997), future studies should consider differences in dietary habits by surveying dietary intake and calculating the nutrient intake on the previous day. Furthermore, BMI and peak oxygen consumption ( $\dot{V}$ O_2_ peak) may also influence baPWV after exercise (Li et al., 2022). Therefore, it was initially necessary to match BMI and $\dot{V}$ O_2_ peak between for females and TM participants. In this study, the criterion was set with BMI >30 kg/m^2^. However, in the future, BMI and $\dot{V}$ O_2_ peak should be made to align both BMI and $\dot{V}$ O_2_ peak levels more closely between the two groups. Aerobic exercise has been shown to decrease atherosclerosis by producing NO (Green et al., 2017). However, this aspect was not measured in the present study. Therefore, in future research, the acute motor response to NO should be examined to gain a clearer understanding of its potential relevance to PWV changes. Considering the above factors, future investigations will need to examine large data sets with larger numbers of participants or longitudinal studies that follow participants before and after treatment. Based on the results of the present study, we suggest that future exercise therapy aimed at reducing arterial stiffness in TM individuals should take into account exercise frequency and also explore the long-term effects of the intervention.

## 5 Conclusion

The present findings suggest that acute AE does not reduce arterial stiffness in TM individuals who undergo TRT. However, acute AE of the TM has been shown to yield different results in males. Future studies are needed to investigate the impact of long-term exercise interventions on arterial stiffness and to prescribe TM undergo TRT with exercise interventions to reduce disease risk.

## Data Availability

The raw data supporting the conclusion of this article will be made available by the authors, without undue reservation.
